# Transcriptomic profiling of clear cell renal cell carcinoma reveals age-dependent molecular signatures and clinical stratification patterns

**DOI:** 10.1371/journal.pone.0344424

**Published:** 2026-03-10

**Authors:** Yun Niu, Yunchao Su, Xiaoxi Wang, Xiaoyuan Yang, Dingrong Zhong

**Affiliations:** 1 Department of Pathology, China-Japan Friendship Hospital, Beijing, China; 2 Department of Pathology, Qinghai Provincial People’s Hospital, Xining, China; Ondokuz Mayis University Faculty of Medicine: Ondokuz Mayis Universitesi Tip Fakultesi, TÜRKIYE

## Abstract

Clear cell renal cell carcinoma (ccRCC) represents the most prevalent form of kidney cancer, yet age-related molecular heterogeneity remains poorly characterized in clinical specimens. We performed comprehensive transcriptomic profiling of 73 formalin-fixed paraffin-embedded (FFPE) ccRCC samples using RNA sequencing to investigate age-dependent molecular signatures and their clinical implications. Principal component analysis (PCA) revealed that PC1 significantly separated younger versus older patients (p = 0.04), while PC2 distinguished tumors by gender (p = 0.00012), size (p = 8 × 10 ⁻ ⁴), and histological class (p = 0.043), suggesting an orthogonal aging molecular axis alongside with disease progression. Differential gene expression analysis identified 330 age-associated genes, with elderly patients showing upregulation of immune checkpoint regulators (CD70), apoptosis modulators (DEDD, HTATIP2), and proton pump components (TCIRG1), alongside downregulation of metabolic enzymes (DIO2) and cytoskeletal regulators (MICALL2). Pathway enrichment analysis revealed dysregulation of aldosterone-regulated sodium reabsorption, B cell receptor signaling, and Th17 cell differentiation pathways, reflecting age-related immunometabolic reprogramming. Integrative analysis of DEGs across clinical variables identified 1,536 shared genes between tumor size and stage comparisons, with CK7-positive tumors exhibiting distinct transcriptional profiles potentially representing a novel molecular subtype. These findings demonstrate that aging fundamentally alters the ccRCC transcriptome through coordinated changes in immune surveillance, metabolic homeostasis, and tumor microenvironment composition, providing a molecular framework for age-stratified therapeutic approaches and precision oncology strategies in renal cell carcinoma.

## Introduction

Clear cell renal cell carcinoma (ccRCC) is the most common subtype of kidney cancer, accounting for approximately 75–80% of all renal malignancies [1]. It arises from the epithelial cells of the proximal tubules and is characterized histologically by clear cytoplasm and a rich vascular network [2]. Clinically, ccRCC exhibits highly variable behavior—while some tumors remain indolent, others progress rapidly, even when organ-confined [3]. Approximately 30% of patients present with metastasis at diagnosis, and recurrence occurs in a substantial subset following nephrectomy [4]. Despite the approval of targeted therapies and immune checkpoint inhibitors, clinical outcomes remain heterogeneous and often unpredictable.

The molecular landscape of ccRCC is defined by frequent inactivation of the von Hippel–Lindau (VHL) tumor suppressor gene, resulting in stabilization of hypoxia-inducible factors (HIFs) and subsequent upregulation of angiogenic, metabolic, and immune-related pathways. CAIX, a downstream target of HIFs, is overexpressed in nearly all ccRCC cases and is widely used as a diagnostic biomarker. In addition to VHL, recurrent mutations in genes such as PBRM1, SETD2, and BAP1—often located on the same 3p chromosomal arm—highlight the contribution of chromatin remodeling and epigenetic regulation to tumor biology. These alterations influence not only tumor growth but also prognosis and therapeutic response.

Transcriptomic profiling, particularly using RNA sequencing (RNA-seq), has been instrumental in elucidating the biological heterogeneity of ccRCC [5]. Gene expression data can distinguish molecular subtypes associated with differential survival, therapeutic vulnerabilities, and immune landscapes [6]. For instance, the widely recognized ccA/ccB classification divides tumors into angiogenic (ccA) and proliferative/inflammatory (ccB) groups, with implications for prognosis and treatment stratification [7]. Studies from The Cancer Genome Atlas (TCGA) have extended this framework by identifying additional expression clusters and linking transcriptomic subtypes to mutational profiles and clinical outcomes [8]. However, TCGA primarily includes treatment-naive primary tumors, limiting its applicability to diverse clinical contexts such as metastatic or post-treatment disease.

Formalin-fixed paraffin-embedded (FFPE) tissues are a valuable resource for retrospective transcriptomic studies, but they pose significant technical challenges [9]. RNA extracted from FFPE samples is often highly fragmented (100–200 nucleotides) and chemically modified, with cytosine deamination introducing artifactual variants that can mislead analyses [10]. These issues compromise transcript integrity and reduce the efficiency of standard library preparation protocols, especially those relying on poly(A)-selection. Additionally, RNA yield from FFPE samples is typically low, often below the threshold required for conventional sequencing methods. Recent methodological advances—including template-switching reverse transcription, low-input library preparation, and computational correction algorithms—now enable reliable transcriptome-wide analysis from degraded RNA. Tools such as SMIXnorm and quality metrics like DV200 enhance data integrity, allowing integration of molecular and clinical datasets at scale [11–13]. These advancements have enabled the possibility of performing extensive transcriptome-wide analyses on FFPE samples.

In this study, we apply FFPE-compatible RNA-seq to a cohort of 73 clinically annotated ccRCC samples to investigate transcriptomic correlates of tumor stage, size, age, and CK7 status. Using principal component analysis and differential gene expression, we identify expression patterns linked to clinical phenotypes, with a focus on age-related immune and metabolic shifts and potential biomarkers for disease stratification. Our findings demonstrate the utility of FFPE transcriptomics in refining ccRCC classification and support further integration of RNA-based profiling into clinical research and practice.

## Materials and methods

### Sample collection and processing

FFPE tumor tissue samples were collected from 89 ccRCC patients. The recruitment period for this study was from 06/07/2024 to 03/16/25. Clinical metadata was systematically recorded for each patient, including gender, age (categorized as ≤45 or >45 years), tumor size (classified as small, medium, or large), histological class (A-D), and immunohistochemical expression status of carbonic anhydrase IX (CAIX) and cytokeratin 7 (CK7). Histological classes A-D represent a relabeling of the International Society of Urological Pathology (ISUP) grade groups 1–4 for descriptive purposes [14]. Following initial quality assessment, 73 samples met inclusion criteria and were retained for downstream transcriptomic analysis. All human participants data were obtained with approval from the China-Japan Friendship Hospital Clinical Research Ethics Committee (Ethics Review No. 2024-KY-170). Informed consent was obtained from all participants, and all datasets were fully de-identified.

### RNA extraction and quality control

Total RNA was extracted from FFPE tissue sections using standard protocols optimized for degraded RNA. RNA quality was assessed using the RNA Integrity Number (RIN) and DV200 metrics (percentage of fragments >200 nucleotides), with DV200 values >50% considered acceptable for FFPE-derived material. Samples with insufficient RNA yield (<10 ng) or poor quality metrics were excluded from further analysis. Post-quality control, the median RNA yield was 85 ng (IQR: 42–120 ng), with median DV200 of 58% (range: 42–79%).

### Library preparation and RNA sequencing

RNA sequencing libraries were prepared using protocols specifically designed for FFPE-derived RNA to accommodate fragmentation and chemical modifications. Ribosomal RNA depletion was performed to capture the whole transcriptome, including non-polyadenylated species. Libraries were sequenced on an Illumina platform to generate paired-end reads with sufficient depth for comprehensive transcriptome coverage.

### Data preprocessing and quality assessment

Raw sequencing data were generated from experiments performed between 06/07/2024 and 03/16/25, and accessed for analysis in 03/16/2025. In short, raw sequencing reads underwent comprehensive preprocessing including adapter trimming and quality filtering using fastqc and fastp. Low-quality bases (Phred score <30) and adapter sequences were removed. Processed reads were aligned to the human reference genome (GRCh38) using STAR aligner. Alignment quality metrics including mapping rates and exon/intron distribution were monitored for quality control purposes.

Sample-level quality control included assessment of total read counts, number of detected genes, and principal component analysis to identify potential batch effects and outliers. Samples with fewer than 10 million reads or fewer than 10,000 detected genes were excluded. Gene-level filtering removed lowly expressed genes with fewer than 10 counts across all samples. We employed K-Dense [15], an agentic research system, to assist with literature review, data analysis, and manuscript drafting. All outputs were reviewed and validated by researchers to ensure accuracy and scientific rigor.

### Differential gene expression analysis

Gene expression quantification was performed using standard read counting methods. Count data were normalized using DESeq2’s median-of-ratios method to correct for library size differences between samples. Differential gene expression analysis was conducted using DESeq2, comparing expression levels across various clinical variables including age groups, gender, tumor size categories, histological classes, and biomarker expression status.

Statistical significance thresholds were set at adjusted p-value <0.05 (Benjamini-Hochberg correction for multiple testing) and absolute log₂ fold change >1. A total of 13 pairwise comparisons were performed across the clinical variables, generating comprehensive differential expression profiles for each comparison.

### Principal component analysis

Principal component analysis was performed on normalized expression data to identify major sources of transcriptional variation. The first two principal components (PC1 and PC2) were examined for associations with clinical variables. Statistical significance of PC associations was assessed using appropriate statistical tests, with p-values <0.05 considered significant. Boxplots were generated to visualize PC score distributions across clinical subgroups.

### Pathway enrichment analysis

Functional enrichment analysis was conducted using the Kyoto Encyclopedia of Genes and Genomes (KEGG) database. DEGs from each comparison were subjected to pathway enrichment analysis using clusterProfiler or similar tools. Enrichment significance was determined using false discovery rate (FDR) ≤0.05. Gene Ontology (GO) analysis was also performed to identify enriched biological processes, molecular functions, and cellular components.

### Intersection analysis

UpSet plots were generated to visualize overlapping DEGs across multiple clinical variable comparisons. This analysis identified genes commonly dysregulated across different clinical features and revealed the extent of shared molecular signatures between various comparisons.

### Statistical analysis and visualization

All statistical analyses were performed using R statistical software. Volcano plots were generated to visualize differential expression results, highlighting significantly upregulated and downregulated genes. Heatmaps displayed expression patterns and effect sizes across comparisons. Statistical significance testing for clinical variable associations with principal components employed appropriate parametric or non-parametric tests depending on data distribution.

## Results

### Study overview and sample characteristics

**Fig 1:**
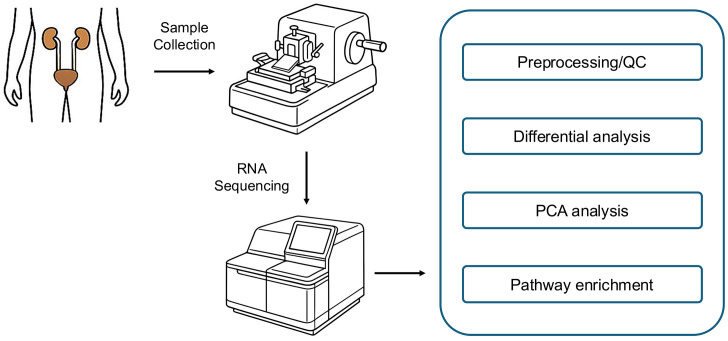
Comprehensive experimental workflow for RNA sequencing analysis of murine kidney tissue samples. The pipeline initiates with sample collection from laboratory mice, specifically targeting kidney tissue (depicted in brown/orange) and associated urogenital structures, followed by tissue processing using rotary microtome sectioning and sample preparation on heating platforms. RNA sequencing is performed using next-generation sequencing technology (represented by high-throughput sequencing instrument with characteristic cabinet design and monitoring display). The bioinformatics analysis pipeline encompasses four sequential computational steps: (1) preprocessing and quality control for raw sequencing data validation, (2) differential gene expression analysis for identifying statistically significant transcriptional changes, (3) principal component analysis (PCA) for dimensionality reduction and sample clustering visualization, and (4) pathway enrichment analysis for functional annotation and biological pathway identification. The workflow demonstrates a complete transcriptomic analysis framework from biological specimen acquisition through computational interpretation, with consistent visual coding distinguishing instrumentation (gray) from computational processing steps (light blue backgrounds).

This transcriptomic analysis examined 73 ccRCC samples derived from FFPE tissue specimens. Fig 1 illustrates the comprehensive experimental workflow, beginning with sample collection from clinical cases and proceeding through RNA sequencing using next-generation sequencing technology. The analytical pipeline encompassed data preprocessing and quality control, differential gene expression analysis, principal component analysis (PCA), and pathway enrichment analysis. Of the initial 89 samples sequenced, 73 samples passed quality control criteria and were retained for downstream transcriptomic analysis.

The cohort characteristics are summarized in Table 1, showing the distribution of samples across key clinical and pathological variables including gender, age groups (≤45 or >45 years), tumor size categories (small, medium, large), histological classes (A-D), and immunohistochemical marker expression status for CAIX and CK7. Specifically, 47 cases were CAIX-positive and CK7-negative, 11 cases were CAIX-positive with focal CK7 positivity, and one case was negative for both markers; notably, no cases exhibited a CAIX-negative and CK7-positive immunophenotype (S1 Table). This sample set provided a representative cross-section of ccRCC cases suitable for comprehensive molecular profiling.

**Table 1:** Summary of clinicopathological features of the included samples. A total of 73 samples were included in the transcriptomic analysis.

Characteristic	Category	n (%)
Gender	Female	21 (28.8%)
	Male	52 (71.2%)
Age (years)	≤45	28 (38.4%)
	>45	45 (61.6%)
Tumor size	Small	43 (58.9%)
	Medium	25 (34.2%)
	Large	5 (6.8%)
Histological class	A	8 (11.0%)
	B	39 (53.4%)
	C	24 (32.9%)
	D	2 (2.7%)
CAIX expression	Positive	59 (80.8%)
	Negative	1 (1.4%)
	N/A	13 (17.8%)
CK7 expression	Positive	11 (15.1%)
	Negative	50 (68.5%)
	N/A	12 (16.4%)

*Abbreviations:* CAIX, Carbonic Anhydrase IX; CK7, Cytokeratin 7; N/A, not available.

### Principal component analysis reveals age-associated molecular signatures

**Fig 2:**
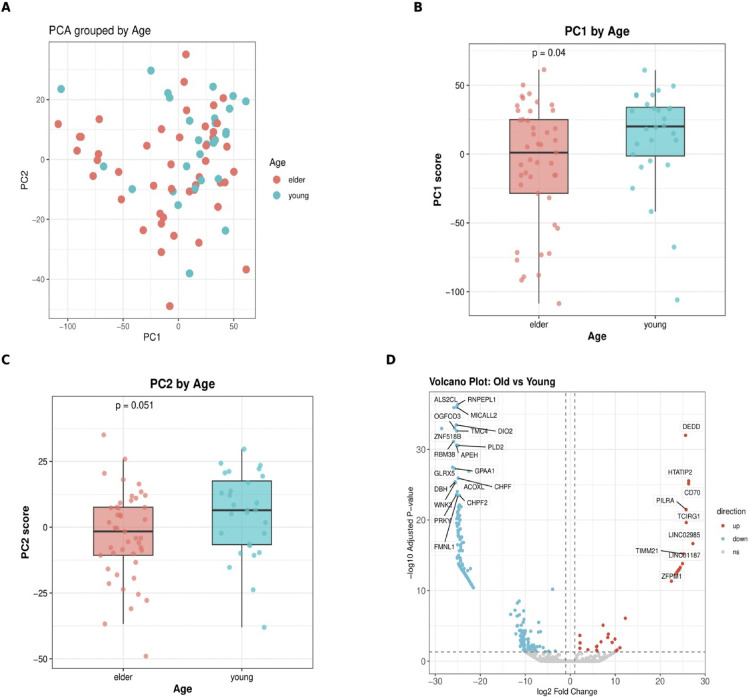
Principal component analysis and differential gene expression analysis reveal age-associated transcriptomic signatures in human subjects. **(A)** Two-dimensional PCA scatter plot displaying PC1 versus PC2 coordinates for individual samples, with elder subjects (salmon circles) and young subjects (teal circles) showing partial age-based clustering, particularly along PC1 where young subjects predominantly occupy higher values (right side of plot). **(B)** Box plot comparison of PC1 scores demonstrates significantly higher values in young versus elder subjects (p = 0.04), with young group median ~+15 and elder group median ~0, indicating PC1 captures primary age-related variance. **(C)** PC2 score distributions show a marginally non-significant trend (p = 0.051) with young subjects exhibiting slightly elevated PC2 values compared to elder subjects. **(D)** Volcano plot of differential gene expression (old vs. young) displays log2 fold change versus -log10 adjusted p-value, with vertical dashed lines marking fold change thresholds (±1 log2) and horizontal line indicating significance cutoff; upregulated genes in elderly include DEDD, HTATIP2, CD70, and PILRA (red points), while downregulated genes include ALS2CL, RNPEPL1, DIO2, and FMNL1 (blue points), demonstrating extensive age-related transcriptional remodeling with PC1 representing the dominant axis of age-associated variation.

Principal component analysis revealed distinct patterns of transcriptomic variation associated with clinical variables, with age emerging as a primary driver of molecular heterogeneity. Fig 2A presents a two-dimensional PCA scatter plot displaying PC1 versus PC2 coordinates, where elder subjects (salmon circles) and young subjects (teal circles) demonstrated partial age-based clustering. Young subjects predominantly occupied higher PC1 values on the right side of the plot, spanning PC1 coordinates from −100 to +50 and PC2 coordinates from −40 to +30.

Statistical analysis of principal component distributions revealed significant age-related differences along PC1. Fig 2B shows box plot comparisons of PC1 scores stratified by age groups, demonstrating significantly higher PC1 values in young versus elder subjects (p = 0.04). The young group exhibited a median PC1 score of approximately +15, while the elder group showed a median near 0, indicating that PC1 captures primary age-related transcriptomic variance. Individual data points overlaid as colored circles confirmed this distribution pattern across the cohort.

PC2 score analysis revealed additional clinical associations beyond age. Fig 2C presents PC2 score distributions showing a marginally non-significant trend (p = 0.051) toward higher PC2 values in young subjects. However, comprehensive statistical testing across all clinical variables demonstrated that PC2 significantly separated samples by gender (p = 0.00012), tumor size (p = 8 × 10 ⁻ ⁴), and histological class (p = 0.043), while showing trends for CK7 expression (p = 0.067). These findings indicate that PC2 represents a secondary axis of variation capturing multiple clinical and pathological features.

### Differential gene expression analysis across clinical variables

Comprehensive differential gene expression analysis across multiple clinical comparisons revealed extensive transcriptomic remodeling associated with various patient characteristics. The DEG summary data showed substantial variation in the number of DEGs across different clinical variables, ranging from hundreds to thousands of genes per comparison.

Fig 2D presents a volcano plot of age-related differential gene expression (old vs. young patients), displaying log2 fold change versus -log10 adjusted p-value. The analysis identified 284 upregulated and 46 downregulated genes in elder patients compared to young patients (total of 330 significant DEGs out of 16,700 genes analyzed). Significantly upregulated genes in elderly patients included DEDD, HTATIP2, CD70, and TCIRG1 (red points), while downregulated genes included ALS2CL, RNPEPL1, MICALL2, and DIO2 (blue points). Vertical dashed lines marked fold change thresholds (±1 log2) and horizontal lines indicated significance cutoffs, with highly significant genes (|log2FC| > 2 and padj < 1e-13) specifically labeled by gene symbol.

**Fig 3:**
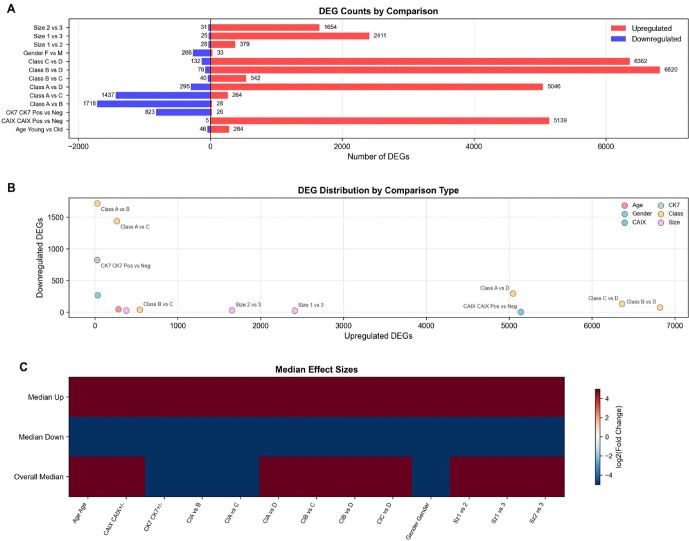
Comprehensive differential gene expression analysis across multiple experimental comparisons reveals distinct transcriptional signatures associated with clinical and biological variables. **(A)** Horizontal bar chart quantifying upregulated (red) and downregulated (blue) DEGs across 13 comparisons, with Class B vs D showing the highest total DEG count (6362 upregulated, 6820 downregulated) and CAX Pos vs Neg exhibiting 5139 upregulated genes. **(B)** Scatter plot correlating upregulated (x-axis, 0-7000) versus downregulated (y-axis, 0-1600) DEGs, color-coded by comparison type (Age, Gender, Class, CK7, CAX, Size), demonstrating Class A vs B with the highest downregulated gene count (~1500). **(C)** Heatmap displaying median log2 fold changes across all comparisons with color scale ranging from −4 (blue) to +4 (red), showing consistent directional expression patterns for upregulated, downregulated, and overall median changes.

The comprehensive DEG analysis across all clinical variables revealed striking differences in transcriptional signatures. Fig 3A quantifies upregulated (red) and downregulated (blue) DEGs across 13 experimental comparisons. The most dramatic differences were observed in histological class comparisons, with Class B vs D showing the highest total DEG count (6,362 upregulated and 6,820 downregulated genes), followed by Class A vs D (5,046 upregulated and 295 downregulated genes). Gender comparisons showed more modest differences (33 upregulated and 266 downregulated genes), while tumor size comparisons demonstrated intermediate levels of differential expression.

Notably, the CAIX expression comparison revealed an extreme transcriptional bias with 5,139 upregulated genes and only 5 downregulated genes in CAIX-positive versus CAIX-negative samples. However, this finding must be interpreted cautiously given the extremely small number of CAIX-negative samples in the cohort, which limits the statistical power and generalizability of this comparison.

To explore factors associated with DEGs, we examined VST-normalized counts across clinical variables (Gender, Age, CAIX status, CK7 status, and histological class) in 73 samples. Interestingly, *CD70* expression was significantly higher in CK7-positive tumors (*p* = 0.026) but showed no significant association with Gender, Age, CAIX status, or histological class (all *p* > 0.05) (S2 Fig A). Given the established biological distinction between Class B and C tumors, we next performed a two-way ANOVA to assess the interaction between histological class and CK7 status on *CD70* expression. CK7 status was identified as a significant main effect (*p* = 0.004), whereas the class × CK7 interaction did not reach statistical significance (*p* = 0.116) (S2 Fig B), likely reflecting limited statistical power given the sample size. Simple-effect analyses based on estimated marginal means indicated that the association between CK7 status and *CD70* expression was driven primarily by Class C tumors (CK7-positive vs. CK7-negative: difference = 3.08, *p* = 0.003), whereas no significant difference was observed within Class B tumors (difference = 1.07, *p* = 0.184) (S2 Fig C). Pairwise comparisons further confirmed that Class C/CK7-positive tumors exhibited significantly higher *CD70* expression than both Class C/CK7-negative (*p* = 0.014) and Class B/CK7-negative (*p* = 0.017) tumors (S2 Fig D). Collectively, these findings suggest that Class C/CK7-positive tumors represent a distinct subgroup characterized by elevated *CD70* expression

### Relationship between upregulated and downregulated gene expression

Fig 3B presents a scatter plot correlating upregulated (x-axis, 0–7,000) versus downregulated (y-axis, 0–1,600) DEGs, color-coded by comparison type (Age, Gender, Class, CK7, CAIX, Size). This analysis revealed distinct patterns in the balance of transcriptional regulation across different clinical variables. Class A vs B comparison demonstrated the highest number of downregulated genes (~1,500), while maintaining a moderate number of upregulated genes, suggesting this comparison captures a predominantly suppressive transcriptional program. In contrast, several comparisons showed predominantly upregulatory patterns, particularly the CAIX and certain class-based comparisons.

The median effect sizes across all comparisons are visualized in Fig 3C as a heatmap displaying log2 fold changes with color intensity ranging from dark blue (−4) to dark red (+4). The heatmap shows three metrics: median upregulated gene fold changes, median downregulated gene fold changes, and overall median changes. This analysis revealed consistent directional expression patterns across experimental conditions, with most comparisons showing balanced upregulation and downregulation patterns, though with varying magnitudes of effect.

### Detailed volcano plot analysis of key comparisons

S1 Fig presents volcano plots for six key comparisons, illustrating the distribution of genes by effect size (x-axis) and statistical significance (y-axis). The age comparison (Young vs Old, Panel A) showed 254 upregulated and 46 downregulated genes, with fold changes spanning −20 to +20 log2 units. The gender comparison (Female vs Male, Panel B) revealed 33 upregulated and 204 downregulated genes with a more constrained fold change range (−10 to +10).

The CAIX comparison (Positive vs Negative, Panel C) displayed the most extreme pattern with 5,139 upregulated and 0 downregulated genes, showing predominantly positive fold changes. This pattern likely reflects the limited number of CAIX-negative samples rather than true biological significance. The Class A vs B comparison (Panel D) exhibited the most balanced pattern with 1,718 upregulated and 1,716 downregulated genes, demonstrating extensive bilateral transcriptional remodeling.

Size-based comparisons (1 vs 2, Panel E) showed 379 upregulated and 29 downregulated genes with moderate fold changes, while CK7 expression comparisons (Positive vs Negative, Panel F) revealed 26 upregulated and 823 downregulated genes, indicating predominantly negative transcriptional regulation associated with CK7 positivity.

### Pathway enrichment analysis

**Fig 4:**
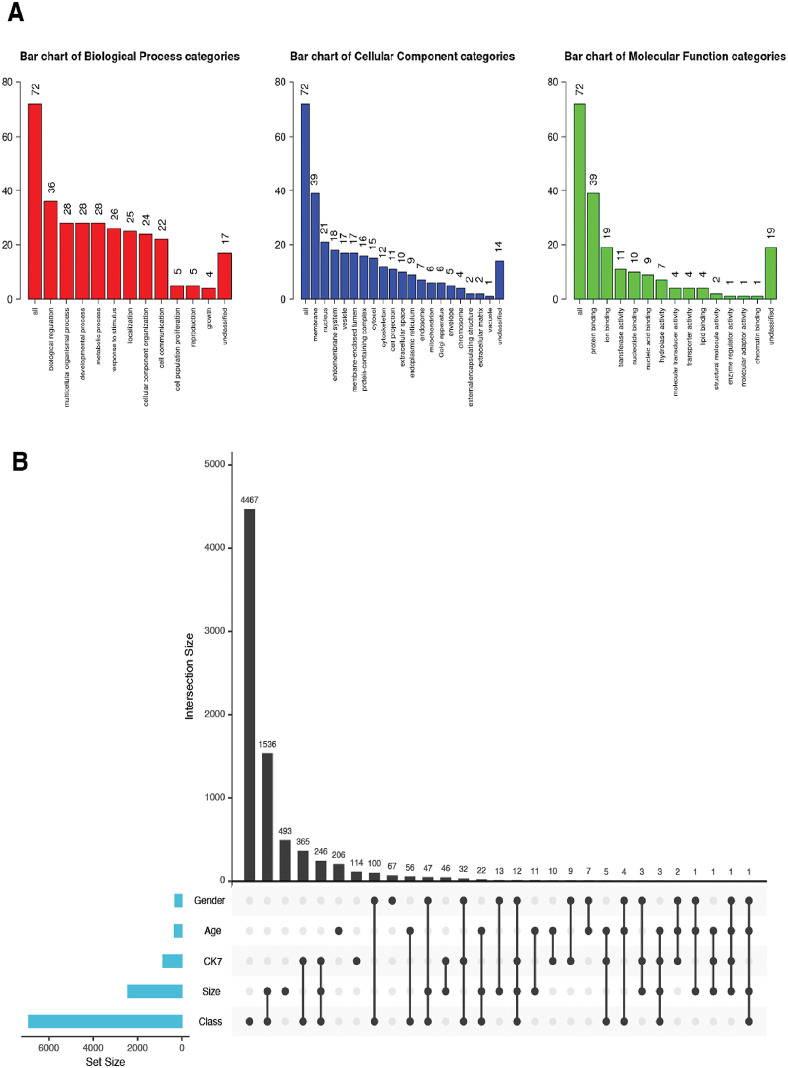
Gene ontology enrichment analysis and categorical variable intersection patterns reveal distinct functional profiles and data structure relationships. **(A)** GO term enrichment analysis across three functional categories shows metabolic processes dominating biological functions (n = 72), with cellular processes (n = 36) and single-organism processes (n = 30) representing secondary enrichment patterns; cellular component analysis demonstrates predominant cell-associated localization (cell: n = 72, cell part: n = 59, organelle: n = 39), while molecular function categories are led by binding activities (n = 72) and catalytic functions (n = 39), indicating a metabolically active cellular profile. **(B)** UpSet plot intersection analysis of five categorical variables (Gender, Age, CK7, Size, Class) reveals a highly skewed distribution with the largest intersection containing 4,467 elements and a secondary major intersection of 1,536 elements, followed by progressively smaller intersections ranging from 493 to single-element combinations, suggesting strong co-occurrence patterns among specific variable combinations. The connection matrix indicates complex multi-variable relationships with varying degrees of overlap across the categorical dimensions, providing insights into the underlying data structure and variable interdependencies.

Gene ontology enrichment analysis revealed distinct functional profiles associated with the differentially expressed genes. Fig 4A presents GO term enrichment across three functional categories. Biological process analysis showed metabolic processes dominating with an enrichment score of 72, followed by cellular processes (36) and single-organism processes (30). Additional significantly enriched biological processes included biological regulation (28), regulation of biological processes (26), localization (25), response to stimulus (24), and signaling (22).

Cellular component analysis demonstrated predominant cell-associated localization patterns, with general cellular components showing the highest enrichment (72), followed by cell parts (59) and organelles (39). Molecular function categories were led by binding activities (enrichment = 72) and catalytic functions (39), indicating a metabolically active cellular profile. These enrichment patterns suggest that age-related transcriptomic changes in ccRCC involve fundamental alterations in cellular metabolism, signaling pathways, and regulatory processes.

### Gene set intersection analysis

Fig 4B presents an UpSet plot showing the intersection of DEGs across multiple clinical and pathological variables, including gender, age, tumor size, histological class, and CK7 expression. The analysis revealed complex patterns of gene overlap across different clinical variables. The largest intersection contained 4,467 genes, followed by a secondary major intersection of 1,536 genes. Subsequent intersections showed progressively smaller sizes ranging from 493 down to single-element combinations.

The horizontal bars represent the total number of DEGs identified for each variable, while the vertical bars indicate the size of intersecting DEG sets across one or more variables. The connection matrix below the main chart shows which specific combinations of variables contribute to each intersection through dot-and-line connectivity patterns. The most substantial overlap was observed between tumor size and histological class (1,536 genes), followed by CK7/Class (365 genes) and CK7/Size/Class (246 genes), suggesting these clinical variables share common underlying molecular mechanisms.

### principal component analysis across multiple clinical variables

**Fig 5:**
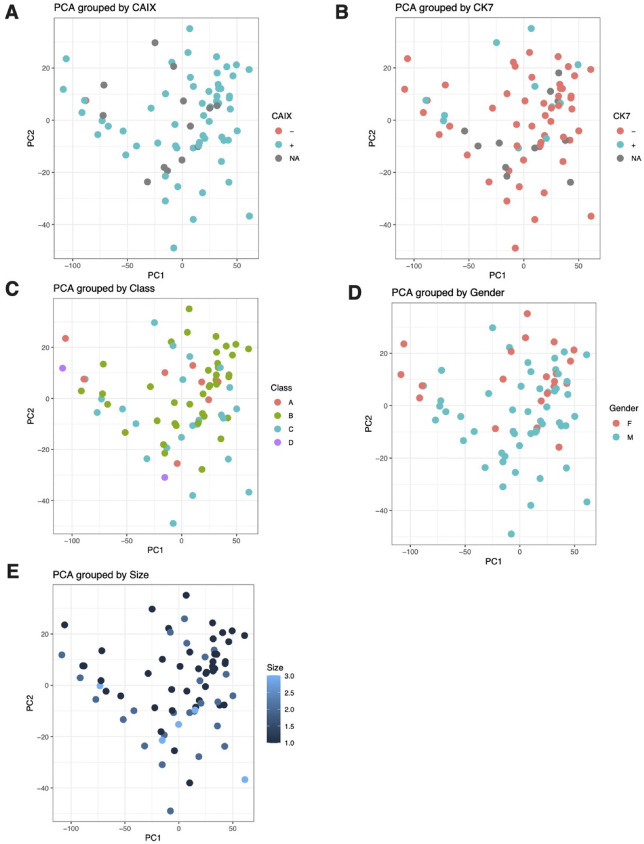
Principal component analysis reveals distinct molecular clustering patterns across biological and clinical variables in tumor specimens (n = 80). **(A)** CAIX expression status segregates samples into three groups: CAIX-negative (teal, n ≈ 45), CAIX-positive (salmon, n ≈ 25), and unavailable data (gray, n ≈ 10), with CAIX-positive samples concentrating in the upper-right quadrant of PC1 vs PC2 space. **(B)** CK7 expression classification shows CK7-negative (teal, n ≈ 35) and CK7-positive (salmon, n ≈ 40) samples with more dispersed distribution patterns compared to CAIX grouping. **(C)** Molecular class stratification identifies four distinct subgroups: Class A (salmon, n ≈ 8), Class B (olive, n ≈ 35), Class C (teal, n ≈ 35), and Class D (purple, n ≈ 4), with Class B demonstrating the broadest PC1 distribution. **(D)** Gender-based analysis reveals overlapping distributions between female (salmon, n ≈ 35) and male (teal, n ≈ 45) samples across principal component space, indicating gender does not drive major dataset variance. **(E)** Tumor size visualization using continuous blue gradient scaling (1.0-3.0 cm) demonstrates size-dependent clustering, with larger tumors preferentially localizing toward positive PC1 values. PC1 and PC2 axes range from −100 to +50 and −50 to +30, respectively, across all panels.

Extended PCA analysis across additional clinical variables provided insights into the molecular basis of ccRCC heterogeneity. Fig 5A shows PCA visualization grouped by CAIX expression status, revealing three distinct groups: CAIX-negative (teal, n ≈ 45), CAIX-positive (salmon, n ≈ 25), and samples with unavailable data (gray, n ≈ 10). CAIX-positive samples showed some concentration in the upper-right quadrant of PC1 vs PC2 space, though with substantial overlap with CAIX-negative samples.

CK7 expression classification (Fig 5B) demonstrated CK7-negative (teal, n ≈ 35) and CK7-positive (salmon, n ≈ 40) samples with more dispersed distribution patterns compared to CAIX grouping. Molecular class stratification (Fig 5C) identified four distinct subgroups: Class A (salmon, n ≈ 8), Class B (olive, n ≈ 35), Class C (teal, n ≈ 35), and Class D (purple, n ≈ 4), with Class B demonstrating the broadest PC1 distribution.

Gender-based analysis (Fig 5D) revealed overlapping distributions between female (salmon, n ≈ 35) and male (teal, n ≈ 45) samples across principal component space, indicating that gender does not drive major dataset variance. Tumor size visualization (Fig 5E) using a continuous blue gradient scaling (1.0–3.0 cm) demonstrated size-dependent clustering patterns, with larger tumors preferentially localizing toward positive PC1 values.

## Discussion

### Transcriptomic signatures and clinical stratification in clear cell renal cell carcinoma

ccRCC exhibits distinct age-dependent molecular signatures that provide insights into tumor biology and clinical outcomes. Principal component analysis (PCA) of transcriptomic data from 73 ccRCC patients demonstrates significant transcriptomic divergence between younger and older patients, with PC1 effectively segregating age groups (p = 0.04) [16]. PC2 correlates with multiple tumor progression parameters including size (p = 8 × 10 ⁻ ⁴), histological class (p = 0.043), and gender (p = 0.00012), establishing a molecular framework for ccRCC subtyping that highlights the complex interplay between aging, tumor biology, and clinical outcomes.

### Age-associated transcriptional remodeling in ccRCC

Transcriptomic analysis of FFPE tissue samples reveals substantial molecular differences between age cohorts in ccRCC. The analysis identified 330 DEGs between younger and older patients, with 284 genes upregulated and 46 genes downregulated in elderly patients. This age-related transcriptional signature reflects fundamental biological processes that become dysregulated with advancing age in the context of renal malignancy.

Key upregulated genes in elderly patients include several functionally significant targets. DEDD, a death effector domain-containing protein, modulates apoptosis resistance and DNA damage response pathways, potentially contributing to enhanced tumor cell survival in older patients [17]. CD70, a member of the TNF superfamily, promotes immunosuppression through T-cell exhaustion mechanisms, creating a more permissive tumor microenvironment for cancer progression [18]. TCIRG1, which encodes a critical component of the vacuolar H + -ATPase complex, enhances extracellular acidification through V-ATPase assembly, facilitating tumor invasion and metastasis [19]. HTATIP2, involved in chromatin remodeling processes, facilitates epigenetic alterations that may contribute to age-related transcriptional dysregulation.

Conversely, genes downregulated in elderly patients include DIO2, which regulates thyroid hormone metabolism and cellular energy homeostasis, ALS2CL, involved in vesicle trafficking and cellular transport mechanisms, and RNPEPL1, which encodes a peptidase with roles in protein processing. This downregulation pattern reflects accelerated immunosenescence and metabolic dysregulation characteristic of aging-associated cancer biology, consistent with established hallmarks of cancer-associated aging [16,17].

The age-related transcriptional changes observed in ccRCC align with broader patterns of cellular senescence and metabolic reprogramming. FFPE-derived RNA sequencing data, despite inherent technical challenges including RNA fragmentation and chemical modifications from formalin fixation, successfully captured these biologically relevant age-associated signatures [20]. Advanced normalization methods such as SMIXnorm have proven effective in correcting FFPE-specific biases, achieving 96.4% concordance with fresh-frozen samples in paired analyses [13].

### Molecular subtyping and biomarker implications

Principal component analysis reveals PC2 as a potential “malignancy axis” that decreases with advanced tumor size and stage while increasing with CK7 positivity (p = 0.067). This finding suggests that PC2 captures a molecular gradient from aggressive, undifferentiated phenotypes (low PC2 values) to more differentiated tumor states (high PC2 values). The differential expression gene intersection analysis demonstrates substantial overlap between tumor size and histological class comparisons, with 1,536 shared DEGs indicating coordinated molecular drivers of progression.

The relationship between clinical variables and molecular signatures is further illustrated by the CK7/Size/Stage triad, which shares 246 DEGs. This overlap suggests that CK7-positive tumors represent a biologically distinct subgroup characterized by preserved epithelial differentiation markers. CK7 expression in ccRCC is typically negative, with focal positivity observed in only 10–15% of cases, making CK7-positive tumors a potentially important molecular subtype for therapeutic stratification [21].

Previous studies have emphasized the molecular heterogeneity and immune-related regulatory programs underlying ccRCC progression and treatment response. Peng et al. identified therapy-associated hub genes linked to immune infiltration and resistance mechanisms, highlighting the importance of tumor–immune interactions in shaping ccRCC biology [22]. Complementary integrative analyses have further demonstrated that immune-associated gene expression patterns, including checkpoint-related markers, correlate with clinical outcomes and may inform therapeutic stratification [23]. Additionally, genetic association studies have identified angiogenesis-related variants, such as vascular endothelial growth factor polymorphisms, that contribute to renal cancer susceptibility [24]. Consistent with these findings, our molecular subtyping analysis reveals clinically relevant transcriptomic heterogeneity associated with tumor differentiation and key clinical features in an age-stratified FFPE cohort.

Carbonic anhydrase IX (CAIX) demonstrates diffuse membranous positivity in 94–97% of ccRCC cases, serving as a diagnostic hallmark of this tumor type [25]. However, the differential expression analysis comparing CAIX-positive versus CAIX-negative samples revealed 5,139 upregulated genes versus only 5 downregulated genes, an extreme transcriptional bias that requires cautious interpretation. This imbalance likely reflects the scarcity of CAIX-negative samples within the cohort, as CAIX negativity is inherently rare in ccRCC biology [25,26]. The minimal representation of CAIX-negative cases reduces statistical power and increases the risk of false discovery, highlighting the importance of balanced sample sizes in biomarker studies.

CD70 is an immune co-stimulatory molecule involved in T cell activation and tumor–immune interactions [27]. In our analysis, elevated CD70 expression was observed primarily in Class C/CK7-positive tumors. This pattern suggests that CD70 upregulation may be context-dependent rather than a general feature of CK7-positive tumors. Although CK7 status showed a significant main effect, the lack of a statistically significant class × CK7 interaction indicates that these subgroup differences should be interpreted cautiously. CK7 positivity is commonly associated with distinct histological characteristics. The enrichment of CD70 expression within the Class C/CK7-positive subgroup may therefore reflect underlying biological differences within this tumor category. However, given the limited sample size, further validation in independent cohorts will be necessary to confirm the robustness and potential biological significance of this association.

### Pathway dysregulation in tumor progression

KEGG pathway enrichment analysis identified four significantly altered pathways that provide insights into the molecular mechanisms underlying ccRCC progression. Aldosterone-regulated sodium reabsorption emerged as the most significantly enriched pathway (enrichment ratio = 4.2, FDR = 0.003), reflecting dysregulation of ion transport mechanisms that parallel VHL-HIF axis alterations characteristic of ccRCC. Aldosterone signaling has been implicated in promoting DNA damage through K-RAS4A activation, with mineralocorticoid receptor antagonists showing therapeutic potential in preclinical models [19].

The enrichment of immune-related pathways, including B cell receptor signaling (enrichment ratio = 3.8, FDR = 0.007), Th17 cell differentiation (enrichment ratio = 3.5, FDR = 0.012), and Fc gamma R-mediated phagocytosis (enrichment ratio = 3.1, FDR = 0.018), indicates significant tumor microenvironment remodeling. These pathways reflect age-exacerbated immunosuppression, consistent with the elevated CD70 expression observed in elderly patients. The immune pathway dysregulation suggests that aging creates a more immunosuppressive tumor microenvironment that may influence treatment response, particularly to immunotherapy approaches [28]. Gene ontology analysis revealed that metabolic processes dominated the biological functions (n = 72), followed by cellular processes (n = 36) and binding activities (n = 72) with catalytic functions (n = 39). This metabolic emphasis aligns with the known metabolic reprogramming that occurs in ccRCC, including the Warburg effect and alterations in oxidative phosphorylation pathways. The prevalence of binding and catalytic activities suggests active enzymatic processes involved in tumor progression and adaptation to the aging cellular environment.

### Clinical translation, therapeutic opportunities and limitations

The age-related differential expression signature offers several actionable targets for precision medicine approaches in ccRCC. The upregulation of CD70 in elderly patients presents a candidate for targeted immunotherapy, particularly given its role in T-cell exhaustion and immune evasion. Anti-CD70 antibodies and other checkpoint blockade strategies may be particularly effective in elderly patients with elevated CD70 expression. Patients with elevated T-effector signatures have shown enhanced correlation with immunotherapy response markers, supporting the potential clinical utility of this approach [18].

Metabolic targeting represents another promising therapeutic avenue. The upregulation of TCIRG1-mediated acidification and suppression of DIO2 create specific metabolic vulnerabilities in elderly ccRCC patients. Inhibitors targeting V-ATPase assembly or thyroid hormone pathway modulators may disrupt tumor survival mechanisms preferentially in elderly cohorts. The metabolic dysregulation observed in aging-associated ccRCC aligns with broader concepts of targeting cancer metabolism as a therapeutic strategy.

Stratification approaches that integrate PC2 values with CK7 status enable molecular subtyping that can predict distinct clinical behaviors. Tumors with low PC2 values and CK7-negative status may represent aggressive phenotypes requiring intensive treatment approaches, while tumors with high PC2 values and CK7-positive status may represent more differentiated subtypes with different treatment sensitivities and resistance patterns.

While our study provides one of the few transcriptomic characterizations of ccRCC based on clinically archived FFPE specimens, enabling direct investigation of age-associated molecular alterations in real-world patient samples, several important limitations should be noted. First, the sample size (n = 73) may limit the generalizability of the results and reduce power to detect subtle changes, particularly in subgroup analyses. Larger multi-center cohorts will be needed to confirm these findings. Second, bulk RNA-seq captures averaged expression across heterogeneous cell populations and cannot resolve cell-type–specific programs or attribute changes to tumor, stromal, or immune compartments. Single-cell or spatial transcriptomic approaches would be required to dissect age-related alterations at higher resolution. Third, the use of FFPE samples may introduce variability in RNA integrity and sequencing coverage despite rigorous quality control, potentially affecting detection of low-abundance transcripts. Finally, we did not perform functional validation of the identified biomarkers or incorporate clinical outcome data. Future studies integrating functional assays and longitudinal clinical follow-up will be essential to establish the biological and clinical relevance of these molecular signatures. Despite these limitations, our findings provide a valuable foundation for understanding age-dependent transcriptomic heterogeneity in ccRCC and highlight specific pathways and biomarkers that warrant further investigation in larger cohorts and mechanistic studies

### Methodological considerations and technical challenges

The analysis of FFPE-derived RNA sequencing data presents unique technical challenges that must be considered when interpreting results. FFPE preservation induces severe RNA fragmentation through formalin-mediated crosslinking, with average fragment sizes of 100–200 nucleotides [29]. This degradation directly compromises transcript integrity and can introduce systematic biases in gene expression measurements. The current study employed whole transcriptome sequencing approaches that enabled detection of 16,700 genes despite moderate fragmentation, demonstrating the feasibility of comprehensive transcriptomic analysis using archival tissue samples.

Chemical modifications introduced during formalin fixation, including cytosine deamination and methylol adduct formation, can generate artifactual sequence changes and reduce cDNA conversion efficiency by 30–60% compared to fresh-frozen samples [30]. Specialized normalization methods and quality control metrics, including DV200 assessment (percentage of fragments >200 nucleotides), are essential for ensuring data reliability [31]. The median DV200 of 58% observed in the current study indicates acceptable RNA quality for transcriptomic analysis.

The extreme differential expression gene imbalance observed in the CAIX analysis (5,139 upregulated versus 5 downregulated genes) exemplifies the potential for overinterpretation when sample sizes are highly imbalanced. This pattern lacks biological plausibility and likely reflects technical artifacts rather than true biological differences. Future studies should employ balanced sampling strategies and multivariate modeling approaches to control for confounding variables and improve statistical validity.

### Implications for personalized medicine and future directions

The integration of transcriptomic profiling with clinical parameters advances personalized approaches in ccRCC management, particularly for aging populations where conventional therapies may show limited efficacy. The age-related molecular signatures identified in this study provide a foundation for developing age-stratified treatment protocols that account for the distinct biological characteristics of tumors in elderly patients.

Spatial transcriptomics approaches, such as GeoMx DSP, offer opportunities to resolve tumor heterogeneity at the tissue level using FFPE samples. These methods can determine whether CK7-positive expression patterns represent true tumor subclones or stromal contamination, addressing important questions about tumor biology and classification. The integration of spatial methods with bulk transcriptomic data could provide more comprehensive insights into tumor microenvironment dynamics and their relationship to aging processes [32].

Future research directions should focus on validating age-related signatures in prospective cohorts using both bulk and single-cell transcriptomic approaches. The investigation of PC2 as a predictive biomarker in therapeutic trials could provide valuable insights into treatment response patterns. Additionally, the exploration of aldosterone signaling inhibitors in elderly ccRCC patients represents a promising therapeutic avenue based on the pathway enrichment findings.

The development of FFPE-specific reference transcriptomes and standardization of pre-analytical variables, including ischemia time limitations and standardized fixation protocols, will improve the reliability and reproducibility of archival tissue-based studies. As these methodological advances mature, the global archive of FFPE tissue blocks will become an increasingly valuable resource for precision oncology research in ccRCC and other malignancies.

The convergence of advanced wet-laboratory methodologies and sophisticated bioinformatic approaches is progressively unlocking the clinical potential of archival tissue specimens for biomarker discovery and molecular subtyping. The findings presented here demonstrate that age-related transcriptomic signatures in ccRCC reflect fundamental biological processes that influence tumor behavior and may guide therapeutic decision-making in clinical practice.

## Supporting information

S1 Table Sample information and CAIX/CK7 immunohistochemical status. (XLSX)

S2 Table The comparison of DEGs analysis at each combination. (XLSX)

S3 Table GO and KEGG enrichment analyses for each comparison of the DEGs analysis. (XLSX)

S1 Fig Volcano plots across multiple experimental comparisons. (PDF)

S2 Fig Association between *CD70* expression, CK7 status, and histological class. (PDF)

## References

[1] Qi X, Li Q, Che X, Wang Q, Wu G. The Uniqueness of Clear Cell Renal Cell Carcinoma: Summary of the Process and Abnormality of Glucose Metabolism and Lipid Metabolism in ccRCC. Front Oncol. 2021;11:727778. doi: 10.3389/fonc.2021.727778 34604067 PMC8479096

[2] Msaouel P, Genovese G, Tannir NM. Renal Cell Carcinoma of Variant Histology: Biology and Therapies. Hematol Oncol Clin North Am. 2023;37(5):977–92. doi: 10.1016/j.hoc.2023.04.019 37244822 PMC11608423

[3] Ross H, Martignoni G, Argani P. Renal cell carcinoma with clear cell and papillary features. Arch Pathol Lab Med. 2012;136(4):391–9. doi: 10.5858/arpa.2011-0479-RA 22458901

[4] Dagher J, Kammerer-Jacquet S-F, Dugay F, Beaumont M, Lespagnol A, Cornevin L, et al. Clear cell renal cell carcinoma: a comparative study of histological and chromosomal characteristics between primary tumors and their corresponding metastases. Virchows Arch. 2017;471(1):107–15. doi: 10.1007/s00428-017-2124-0 28488172

[5] Neely BA, Wilkins CE, Marlow LA, Malyarenko D, Kim Y, Ignatchenko A, et al. Proteotranscriptomic Analysis Reveals Stage Specific Changes in the Molecular Landscape of Clear-Cell Renal Cell Carcinoma. PLoS One. 2016;11(4):e0154074. doi: 10.1371/journal.pone.0154074 27128972 PMC4851420

[6] Barata P, Gulati S, Elliott A, Hammers HJ, Burgess E, Gartrell BA, et al. Renal cell carcinoma histologic subtypes exhibit distinct transcriptional profiles. J Clin Invest. 2024;134(11):e178915. doi: 10.1172/JCI178915 38652565 PMC11142736

[7] D’Aniello C, Berretta M, Cavaliere C, Rossetti S, Facchini BA, Iovane G, et al. Biomarkers of Prognosis and Efficacy of Anti-angiogenic Therapy in Metastatic Clear Cell Renal Cancer. Front Oncol. 2019;9:1400. doi: 10.3389/fonc.2019.01400 31921657 PMC6917607

[8] Cancer Genome Atlas Research Network, Weinstein JN, Collisson EA, Mills GB, Shaw KRM, Ozenberger BA, et al. The Cancer Genome Atlas Pan-Cancer analysis project. Nat Genet. 2013;45(10):1113–20. doi: 10.1038/ng.2764 24071849 PMC3919969

[9] Groelz D, Sobin L, Branton P, Compton C, Wyrich R, Rainen L. Non-formalin fixative versus formalin-fixed tissue: a comparison of histology and RNA quality. Exp Mol Pathol. 2013;94(1):188–94. doi: 10.1016/j.yexmp.2012.07.002 22814231

[10] Steiert TA, Parra G, Gut M, Arnold N, Trotta J-R, Tonda R, et al. A critical spotlight on the paradigms of FFPE-DNA sequencing. Nucleic Acids Res. 2023;51(14):7143–62. doi: 10.1093/nar/gkad519 37351572 PMC10415133

[11] von Ahlfen S, Missel A, Bendrat K, Schlumpberger M. Determinants of RNA quality from FFPE samples. PLoS One. 2007;2(12):e1261. doi: 10.1371/journal.pone.0001261 18060057 PMC2092395

[12] Matsubara T, Soh J, Morita M, Uwabo T, Tomida S, Fujiwara T, et al. DV200 Index for Assessing RNA Integrity in Next-Generation Sequencing. Biomed Res Int. 2020;2020:9349132. doi: 10.1155/2020/9349132 32185225 PMC7063185

[13] Yin S, Zhan X, Yao B, Xiao G, Wang X, Xie Y. SMIXnorm: Fast and Accurate RNA-Seq Data Normalization for Formalin-Fixed Paraffin-Embedded Samples. Front Genet. 2021;12:650795. doi: 10.3389/fgene.2021.650795 33841507 PMC8024626

[14] Epstein JI, Egevad L, Amin MB, Delahunt B, Srigley JR, Humphrey PA, et al. The 2014 International Society of Urological Pathology (ISUP) Consensus Conference on Gleason Grading of Prostatic Carcinoma: Definition of Grading Patterns and Proposal for a New Grading System. Am J Surg Pathol. 2016;40(2):244–52. doi: 10.1097/PAS.0000000000000530 26492179

[15] Orion L, Vinayak A, Summer Z, Ashwin G, Timothy K. K-dense analyst: towards fully automated scientific analysis. arXiv preprint arXiv:2508.07043. 2025.

[16] Jespersen J, Lindgaard C, Iisager L, Ahrenfeldt J, Lyskjær I. Lessons learned from spatial transcriptomic analyses in clear-cell renal cell carcinoma. Nat Rev Urol. 2025;22(11):726–34. doi: 10.1038/s41585-024-00980-x 39789293

[17] Chen Z, Zhou L, Liu L, Hou Y, Xiong M, Yang Y, et al. Single-cell RNA sequencing highlights the role of inflammatory cancer-associated fibroblasts in bladder urothelial carcinoma. Nat Commun. 2020;11(1):5077. doi: 10.1038/s41467-020-18916-5 33033240 PMC7545162

[18] Ransohoff JD, Wei Y, Khavari PA. The functions and unique features of long intergenic non-coding RNA. Nat Rev Mol Cell Biol. 2018;19(3):143–57. doi: 10.1038/nrm.2017.104 29138516 PMC5889127

[19] Heinrich A-K, Lucas H, Schindler L, Chytil P, Etrych T, Mäder K, et al. Improved Tumor-Specific Drug Accumulation by Polymer Therapeutics with pH-Sensitive Drug Release Overcomes Chemotherapy Resistance. Mol Cancer Ther. 2016;15(5):998–1007. doi: 10.1158/1535-7163.MCT-15-0824 26939698

[20] Lin Y-H, Wang L-W, Chen Y-H, Chan Y-C, Hu S-H, Wu S-Y, et al. Revealing intact neuronal circuitry in centimeter-sized formalin-fixed paraffin-embedded brain. eLife. 2024;13. doi: 10.7554/elife.93212.4 PMC11111220 38775133

[21] Kim M, Joo JW, Lee SJ, Cho YA, Park CK, Cho NH. Comprehensive Immunoprofiles of Renal Cell Carcinoma Subtypes. Cancers (Basel). 2020;12(3):602. doi: 10.3390/cancers12030602 32150988 PMC7139472

[22] Peng Y, Dong S, Song Y, Hou D, Wang L, Li B, et al. Key sunitinib-related biomarkers for renal cell carcinoma. Cancer Med. 2021;10(19):6917–30. doi: 10.1002/cam4.4206 34402193 PMC8495283

[23] Lu Y, Song Y, Xu Y, Ou N, Liang Z, Hu R, et al. The prevalence and prognostic and clinicopathological value of PD-L1 and PD-L2 in renal cell carcinoma patients: a systematic review and meta-analysis involving 3,389 patients. Transl Androl Urol. 2020;9(2):367–81. doi: 10.21037/tau.2020.01.21 32420142 PMC7215048

[24] Song Y, Hu J, Chen Q, Guo J, Zou Y, Zhang W, et al. Association between vascular endothelial growth factor rs699947 polymorphism and the risk of three major urologic neoplasms (bladder cancer, prostate cancer, and renal cell carcinoma): A meta-analysis involving 11,204 subjects. Gene. 2018;679:241–52. doi: 10.1016/j.gene.2018.09.005 30195633

[25] Leibovich BC, Sheinin Y, Lohse CM, Thompson RH, Cheville JC, Zavada J, et al. Carbonic anhydrase IX is not an independent predictor of outcome for patients with clear cell renal cell carcinoma. J Clin Oncol. 2007;25(30):4757–64. doi: 10.1200/JCO.2007.12.1087 17947723

[26] Pastorekova S, Gillies RJ. The role of carbonic anhydrase IX in cancer development: links to hypoxia, acidosis, and beyond. Cancer Metastasis Rev. 2019;38(1–2):65–77. doi: 10.1007/s10555-019-09799-0 31076951 PMC6647366

[27] Wu R, Chen J, Wang G, Han L. CD70 as a target in cancer immunotherapy: advances, challenges, and future directions. Front Oncol. 2025;15:1609840. doi: 10.3389/fonc.2025.1609840 40896423 PMC12394045

[28] Oh E, Kim J-H, Um J, Jung D-W, Williams DR, Lee H. Genome-Wide Transcriptomic Analysis of Non-Tumorigenic Tissues Reveals Aging-Related Prognostic Markers and Drug Targets in Renal Cell Carcinoma. Cancers (Basel). 2021;13(12):3045. doi: 10.3390/cancers13123045 34207247 PMC8234889

[29] Adiconis X, Borges-Rivera D, Satija R, DeLuca DS, Busby MA, Berlin AM, et al. Comparative analysis of RNA sequencing methods for degraded or low-input samples. Nat Methods. 2013;10(7):623–9. doi: 10.1038/nmeth.2483 23685885 PMC3821180

[30] Graw S, Meier R, Minn K, Bloomer C, Godwin AK, Fridley B, et al. Robust gene expression and mutation analyses of RNA-sequencing of formalin-fixed diagnostic tumor samples. Sci Rep. 2015;5:12335. doi: 10.1038/srep12335 26202458 PMC4511951

[31] Li P, Piao Y, Shon HS, Ryu KH. Comparing the normalization methods for the differential analysis of Illumina high-throughput RNA-Seq data. BMC Bioinformatics. 2015;16:347. doi: 10.1186/s12859-015-0778-7 26511205 PMC4625728

[32] Xiao G, Zhang X, Zhang X, Chen Y, Xia Z, Cao H, et al. Aging-related genes are potential prognostic biomarkers for patients with gliomas. Aging (Albany NY). 2021;13(9):13239–63. doi: 10.18632/aging.203008 33946049 PMC8148480

