# Supplementary material for: Transcriptomic profiling of clear cell renal cell carcinoma reveals age-dependent molecular signatures and clinical stratification patterns
Source: PLoS One. 2026 Mar 10;21(3):e0344424. doi: 10.1371/journal.pone.0344424 (PMC12974835; doi:10.1371/journal.pone.0344424)
Supplement: S1 Fig — (PDF) [file pone.0344424.s004.pdf]

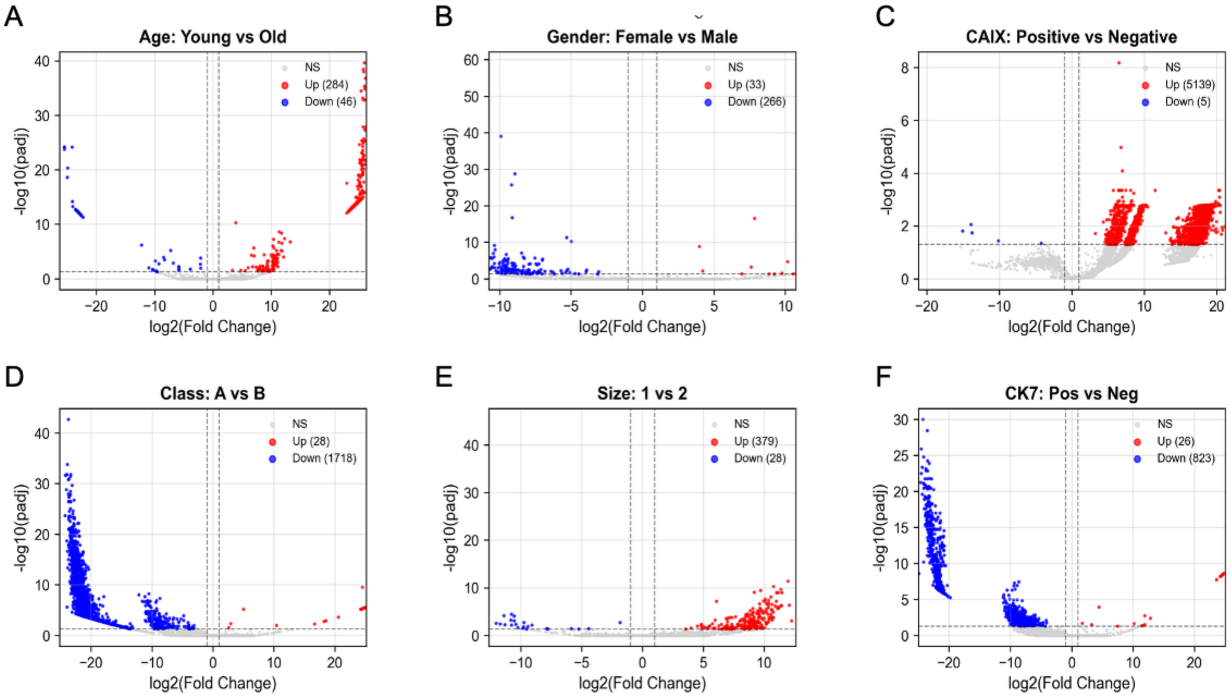

S1 Fig. Volcano plots across multiple experimental comparisons. Volcano plots illustrating statistical significance (-log10 padj, y-axis) versus log2 fold change (x-axis) for six key comparisons: Age Young vs Old (254 up, 46 down), Gender Female vs Male (33 up, 204 down), CAIX Positive vs Negative (5139 up, 0 down), Class A vs B (1718 up, 1716 down), Size 1 vs 2 (379 up, 29 down), and CK7 Positive vs Negative (26 up, 823 down). Significant genes are colored red (upregulated) and blue (downregulated) with gray indicating non-significant changes, while dashed lines denote statistical significance and fold change thresholds.
