# Supplementary material for: Transcriptomic profiling of clear cell renal cell carcinoma reveals age-dependent molecular signatures and clinical stratification patterns
Source: PLoS One. 2026 Mar 10;21(3):e0344424. doi: 10.1371/journal.pone.0344424 (PMC12974835; doi:10.1371/journal.pone.0344424)
Supplement: S2 Fig — (PDF) [file pone.0344424.s005.pdf]

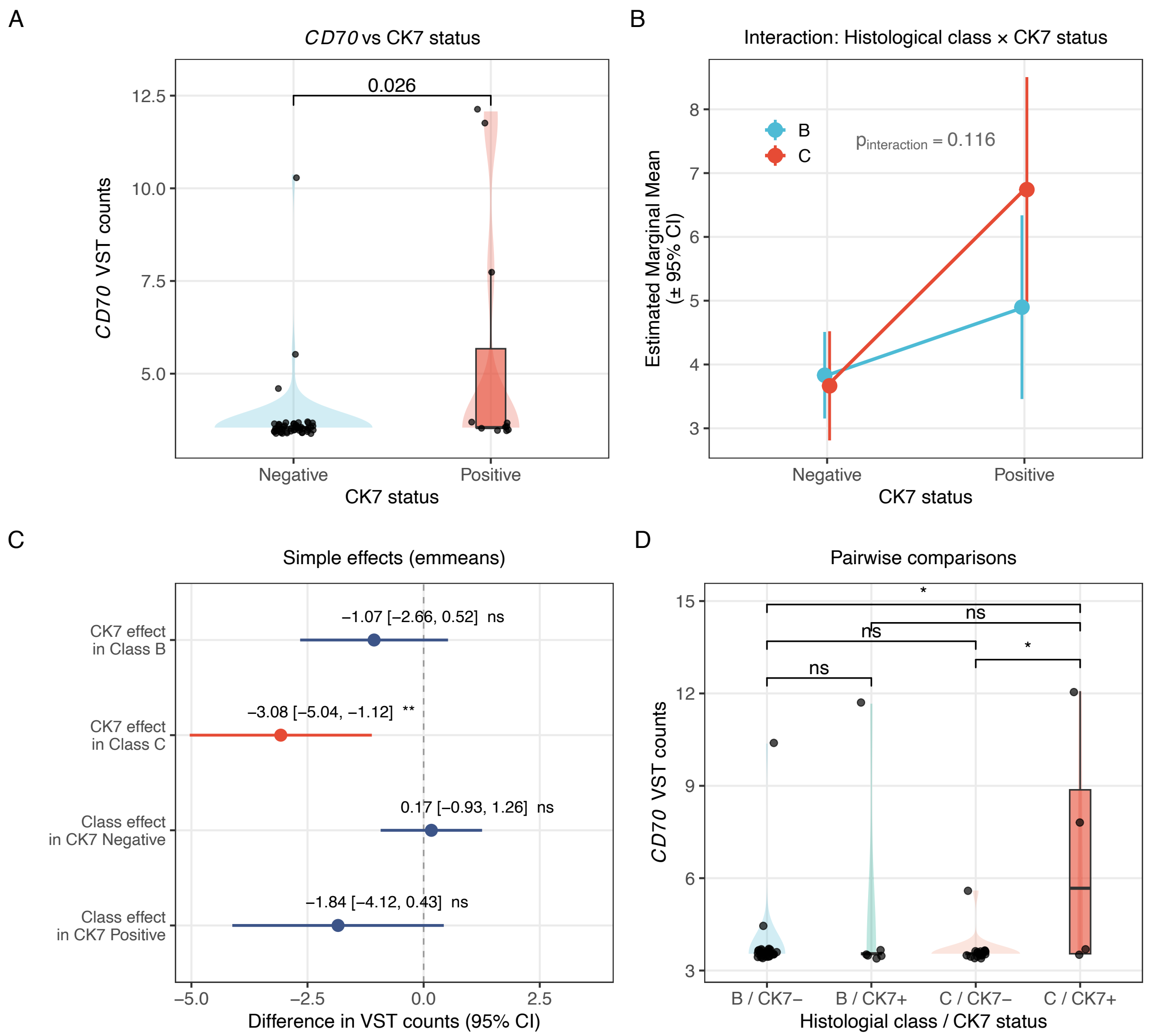

S2 Fig. Association between *CD70* expression, CK7 status, and histological class. (A) *CD70* VST-normalized counts stratified by CK7 status. (B) Two-way ANOVA evaluating the interaction between histological class (B vs. C) and CK7 status on *CD70* expression. Points represent estimated marginal means with 95% confidence intervals. (C) Simple-effect analyses based on estimated marginal means showing the effect of CK7 status within each histological class and the effect of histological class within each CK7 subgroup. (D) Pairwise comparisons of *CD70* expression across combined histological class and CK7 subgroups. Statistical significance is indicated as *$P < 0.05$; **$P < 0.01$; ns, not significant.
